# Quantifying Geographic Variation in the Climatic Drivers of Midcontinent Wetlands with a Spatially Varying Coefficient Model

**DOI:** 10.1371/journal.pone.0126961

**Published:** 2015-04-27

**Authors:** Christian Roy

**Affiliations:** 1 Faculté de foresterie, de géographie et de géomatique and Centre d’étude de la Forêt, Université Laval, Pavillon Abitibi-Price, Québec, Canada; 2 Centre d’étude de la forêt, Université Laval, Pavillon Abitibi-Price, Québec, Canada; Ecologie, Systématique & Evolution, FRANCE

## Abstract

The wetlands in the Prairie Pothole Region and in the Great Plains are notorious for their sensitivity to weather variability. These wetlands have been the focus of considerable attention because of their ecological importance and because of the expected impact of climate change. Few models in the literature, however, take into account spatial variation in the importance of wetland drivers. This is surprising given the importance spatial heterogeneity in geomorphology and climatic conditions have in the region. In this paper, I use spatially-varying coefficients to assess the variation in ecological drivers in a number of ponds observed over a 50-year period (1961-2012). I included the number of ponds observed the year before on a log scale, the log of total precipitation, and mean maximum temperature during the four previous seasons as explanatory variables. I also included a temporal component to capture change in the number of ponds due to anthropogenic disturbance. Overall, fall and spring precipitation were most important in pond abundance in the west, whereas winter and summer precipitation were the most important drivers in the east. The ponds in the east of the survey area were also more dependent on pond abundance during the previous year than those in the west. Spring temperature during the previous season influenced pond abundance; while the temperature during the other seasons had a limited effect. The ponds in the southwestern part of the survey area have been increasing independently of climatic conditions, whereas the ponds in the northeast have been steadily declining. My results underline the importance of accounting the spatial heterogeneity in environmental drivers, when working at large spatial scales. In light of my results, I also argue that assessing the impacts of climate change on wetland abundance in the spring, without more accurate climatic forecasting, will be difficult.

## Introduction

Wetlands in the Prairie Pothole Region (PPR) and the Great Plains (GP) region of North America occur in small depressions (potholes) formed during the last glaciation. Most potholes are small (< 1 ha). They are highly diverse in shape, depth, and deposits, which makes them collectively one of the most diverse inland wetlands in North America [1]. Individual potholes are generally hydrologically isolated and have underlying deposits of low permeability [2], which make them highly dependent on precipitation for their water supply [3]. The climate in the middle of the continent is characterised by large inter-annual variation and extreme weather events [4], and the dependence of wetlands on climatic conditions induces an irregular sequence of wet periods and drought that is one of the defining features of the region [5].

The dynamic nature of the potholes combined with their shallowness, allows them to heat up quickly in the spring, which in turn makes them highly productive [6,7]. In consequence, they are intensively used as breeding habitat by many species of waterfowl and waterbirds. They also represent a staging area for many migrating species [8–10]. In fact, for many duck species, the size of the breeding population is closely correlated to the state of midcontinental wetlands [11], which have accordingly received considerable attention [12–14]. Much of this attention stems from the fact that wetland numbers have dwindled over time (converted to agricultural land uses [15–17]) but also because the pothole system is expected to be very sensitive to climate change [18,19].

However, relatively little attention has been given to the spatial variation in the drivers of wetland dynamics (see [19]). Most previous studies have used some form of variable selection to identify a small set of useful predictors of wetland dynamics, which are then used to predict or forecast over a vast region. The neglect of spatial heterogeneity in describing wetlands dynamics is surprising given the importance in variation in land use, geomorphology, and climatic conditions have in the region. There is a strong north-south temperature and east-west precipitation gradient that produces regionally distinct wet-drought cycles (S1 Fig) [18]. The wetlands at the northern and eastern limits of the Prairies are also thought to be more resilient to drought because they are deeper [15,20]. Heterogeneity in factors, such as agricultural activity and primary productivity, can lead to variation in wetland abundance [21]. Human land uses, including draining, can also cause permanent wetland loss. Rates of wetland loss also vary spatially, being higher in the east than in the west [16,17].

Ecologists have long viewed spatial heterogeneity as an unwelcomed complication [22,23]. While ecologists have started to embrace space as a source of information [24], most spatial models focus on spatial autocorrelation [25]. Conventional regression methods generally estimate a single parameter for each explanatory variable, and the relationship is therefore assumed to be stationary (e.g. constant throughout the region of interest). Unfortunately, relationships are often not stable in space, and it is unreasonable to expect relationships to be stationary over large geographic areas [26,27]. Non-stationarity can be induced by sampling variation, a misspecified model, such as missing variables, or a misspecified functional form, or an intrinsically different process occurring in different parts of the study area [28]. In the presence of a non-stationary relationship, using single regression parameters could be misleading since they will represent the average tendency in the dataset. Under these circumstances, it is quite possible that the parameters derived from a global model would not represent the true relationship anywhere within the region of study [26]; in a worst-case scenario, the use of a single parameter, with trends acting in different directions across the study area, could even lead to Simpson’s paradox [29]. A situation in which the trend identified in a subset of data is the opposite from that identified in the complete dataset.

Since spatial non-stationarity is more difficult to understand and resolve compared to spatial autocorrelation, it has received less attention [30]. However, two broad families of linear models have been recently proposed to deal with spatial non-stationarity: geographically-weighted regression (GWR) and spatially-varying coefficients (SVC). GWR consist of an ensemble of local regressions that are fitted separately [28], whereas SVC models entail only a single hierarchical model [31–33]. While GWR have seen a surge in popularity, the SVC approach is now increasingly applied [34,35]. The definition of a single global model can be seen as advantageous, since it allows the sharing of information across locations [36]. Moreover, SVC models have been proven to be more robust to collinearity among predictors than GWR, which can be an important advantage [35].

In this paper I use the SVC approach to assess the spatial variability in the climatic drivers of wetland dynamics in the midcontinent of North America. My primary objectives were to (a) assess the proportion of variance, in the abundance of wetlands observed during the spring, due to changes in seasonal precipitation and temperature during the previous year, and (b) to quantify the spatial variation in related processes. A secondary objective was to assess if the quantity of wetlands have changed over time in the survey area, independent of climatic drivers.

## Material and Methods

### Pond data

**Fig 1:**
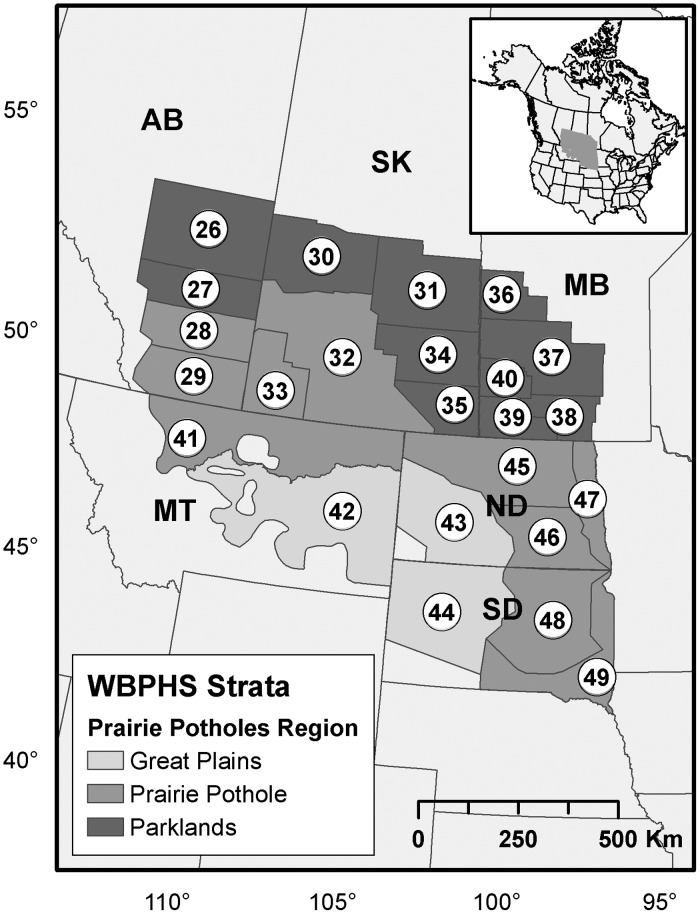
Survey area. Map of the Waterfowl Breeding Population and Habitat Survey area with numbers indicating survey strata used in the analyses (n = 24); AB = Alberta, SK = Saskatchewan, MB = Manitoba, MT = Montana; ND = North Dakota; SD = South Dakota.

Wetland count data were obtained from the Waterfowl Breeding Population and Habitat Survey (WBPHS), conducted annually since 1955 by the U.S. Fish and Wildlife Service and the Canadian Wildlife Service. The WBPHS is a systematic stratified survey of breeding waterfowl and wetlands over a large section of the major waterfowl breeding areas in Canada and the United States of America (USA). The survey takes place between late May and early June. Surveys are flown by fixed wing aircraft on pre-determined transects at speeds of approximately 190 km h^-1^ at altitudes of 30–50 m above ground level. The pilot and an observer count all ducks and wetlands (henceforth referred to as ‘pond’) observed within 200 m on either side of the flight line. The ponds included in the counts are either natural or artificial ponds that are flooded seasonally, semi-permanently and permanently; temporary wetlands that are not likely to persist for 3 weeks beyond the survey date and sheetwater are not counted. Aerial inventories are corrected for visibility by using ground survey data that are conducted concurrently at a subsample of the aerial survey transects (for more details, see [37]). The total abundance of ponds in the survey area are subsequently derived from visually-corrected values. I used the total annual abundance of ponds for the strata associated with the Parkland, Prairie Pothole and Grassland Regions (Fig 1). I restricted the time period to 1961 through 2012 to ensure overlap between pond and annual weather data.

### Explanatory variables and Expectations

In modelling annual pond counts, past studies suggested that total seasonal precipitation and temperature, in all four seasons preceding the survey in May, be included as covariates, as well as the number of ponds in the previous year [12–14,38]. I expected: lagged pond counts to have a positive effect because of a holdover in water conditions [12,13]; precipitation to have a positive effect; and temperature to have a negative effect [12,14,38]. More precisely, I expected winter and fall precipitation to have the largest contribution to the abundance of ponds, because water is more likely to accumulate in the wetland during those seasons, and summer and spring precipitation to have a more modest contribution [12,39]. The effect of temperature should be more important in the spring, followed by fall, and be of lesser importance in summer and winter [12]. Spatially, I expected that precipitation would be more important in the strata associated with the Prairie Pothole Region (PPR), since the strata in the east receive more precipitation that those in the west [18,40] (S1 Fig). For the analysis, I used the log transformed values of the number of ponds during the previous year, and of the total precipitation, to capture the idea that there should be a threshold in their effect. For example, an increase of precipitation at low values would have a stronger effect on pond abundance than at high values.

I also included a linear effect of time, to capture any systematic change, or non-stationarity, in the number of ponds, independent of climatic variables. There has been considerable loss of wetlands in the survey area since settlement because wetlands have been drained and filled for agriculture [17,40]. While the current wetlands loss are still due in great part to agriculture, the expansion of urban areas and transportation corridors are also increasingly important [41]. This has led to many dedicated programs to restore and conserve wetlands in Canada and in the USA [14,15]. Annual rate of wetland area loss in the PPR of Canada was estimated to be around 0.31% a year between 1985 and 2001 [16]. This rate was apparently the same as in previous decades, although there was considerable spatial variation within the PPR [16]. Based on results from Watmough and Schmoll [16], I expected the pond counts in Canadian strata to decrease over the study period and the rate of decrease to be highest in the strata in northeastern Saskatchewan and in Alberta. For the USA strata, the current information available is conflicting. A study looking at the rate of wetland loss in the region indicated that although the current wetland loss abated during the last decades, the rate of decline due to anthropogenic activities is about 0.3 to 0.4% a year [17,42–44]. Conversely, a recent analysis of this region's pond data indicated an increase in pond abundance [14]. However, this study did not take into account the potential role of climatic drivers in observed increases in pond abundance. Given that the amount of precipitation has increased during the latter part of my study period [5] and that there are still wetland losses due to anthropogenic activities, I expected a declining trend over the study period in the U.S. strata.

### Climatic data

I calculated mean seasonal temperatures and total precipitation for each strata and year for summer (June-August), fall (September-November), winter (December-February), and spring (March-May). The source data were estimates of monthly precipitation and monthly means of daily maximum temperature, interpolated to a 300 arc second grid using the ANUSPLIN software package, which employs thin plate smoothing splines to develop elevation-dependent climate surfaces from weather station data. A detailed description of the methodology can be found in McKenney et al. [45]. For the analysis, the mean temperature and total precipitation grid-cell level values were aggregated within strata and seasons using PostGIS 2.0 [46].

### Model

The pond dataset had only positive values, a skewed distribution with a median smaller than the mean, and showed heteroscedasticity among strata. I therefore use a lognormal distribution to model pond dynamics over time [21,47] (Eq 1).

$${\text{Pond}}_{s,t}\sim \text{LN}\left(\mu_{s,t},\tau_{s}\right)$$ (1)

where Pond is total abundance of ponds estimated in strata *s* at time *t*, *μ*_*s*,*t*_ is the expected number of ponds on a natural logarithmic scale and *τ*_*s*_ is the standard deviation that accommodates the unexplained variation in the model on a natural logarithmic scale. The expected number of ponds on the log scale is itself dependent on the explanatory variables:

$$\mu_{s,t}=\;\;XΒ+\text{log}\left({\text{area}}_{s}\right)$$ (2)

where **X** is a three-dimensional array containing the explanatory variables *p* for each strata *s* at time *t*. The first entry in the matrix of explanatory variables is a vector of 1 to accommodate the intercept.**B** is a matrix where each row holds a vector of predictor *p* for each strata *s*. I use the log of the strata area as an offset to account for the variation in strata area [48]. I modeled the spatial variation in the coefficients as a mean effect *μ*_*β*_ and an additive strata-level random effect *w*_*βs*_, which is the local deviation from the mean. This approach can be seen as an extension of the standard random effect model, where the spatial dependence between the parameters is modelled explicitly [33]. This is done via a spatial covariance matrix which defines the local variance and spatial correlation structure of the random effects.

The covariance matrix on the spatial error can have either a separable or nonseparable form [33,34]. The difference between the two forms of specification depends on the specification of the spatial covariance structure. For the non-separable form, each parameter has an independent spatial parameter. While more flexible, this approach is much more computationally difficult [34,35]. The separable approach assumes that explanatory variables have a common spatial dependence structure and can account for autocorrelation among the predictors [33]. It is generally inappropriate to assume independence between the predictors, so it is commonly suggested to use the separable form [33]. For my model, I used a model that represents a compromise between these two extremes by applying a different correlation matrix to the intercept than what was applied to the explanatory variables. The intercept represents the mean abundance of potholes in the landscape. This is a consequence of geomorphological process, such as the glacier activity during the Wisconsin glaciation [40], and it is doubtful that the process that led to the creation of potholes in the landscape in the past would have a similar dependence structure that the ones that govern the current relationship between the predictors and pond abundance. I preferred to use a separable form of the SVC model for the explanatory variables since they are mostly composed of climatic variables, and while the factors that govern the relationship between these predictors and pond abundance could be different, they are not likely to be independent. The intercept is modeled as a constant plus a strata-specific random effect (Eq 3)

$$\;\beta_{0,s}=\;\mu_{0}+{\text{w}}_{0,s}$$ (3)

$$w_{0}{}_{s}\sim \text{MVN}\left(0,\sigma_{\beta_{0}}^{2}{\text{K}}_{0}\right)$$ (4)

The spatial error was therefore modelled as a multivariate process, where $\sigma_{\beta_{0}}^{2}$ represents the variance associated with the intercept, and **K**_0_ is a spatial correlation matrix for the spatial dependence of the intercept among strata. For the correlation function, I used an isotropic Gaussian spatial dependence function $\left({\text{K}}_{0_{i,j}}={\text{e}}^{-\phi^{2}*{\left|d_{i,j}\right|}^{2}}\right)$ based on a preliminary explanatory analysis of the spatial dependence in pond abundance across the study area. For the explanatory variables, the mean effects are estimated independently, but the spatial errors are estimated via a matrix normal distribution, which is a generalisation of the multivariate distribution.

$$w_{s,p}\sim \text{ MN}\left(0_{s,p},{\text{K}}_{Β},\text{T}\right)$$ (5)

where **w** is the error matrix that is indexed by strata *s* and parameter *p*, **K**_B_ is a correlation matrix that assess the spatial dependence among sites (i.e, the rows), and **T** is variance-covariance matrix that assesses the correlation among the coefficients themselves (i.e. the columns). The diagonal elements of **T** are the spatial variance of the parameters. If the need arises, the matrix normal distribution can be vectorized and recast into a multivariate normal distribution, such as:

$$w_{ip}\sim \text{MVN}\left(0,{\text{Σ}}_{Β}\right)$$ (6)

$${\text{Σ}}_{Β}=\text{K}⊗\text{T}$$ (7)

where ⊗ denotes the Kronecker product of the two matrices, and **Σ** is a strata × parameters by strata × parameters matrix. I used a Gaussian spatial dependence function for the spatial correlation matrix similar to the one I used for the intercept. For the variance-covariance function among the parameters, I used a separation approach where the standard deviation for each parameter (σ_*β*_) and the correlation matrix among parameters (*ρ*) were estimated independently.

### Model estimation

Bayesian models need the assignment of a prior distribution for each of the parameters (*μ*_*β*_, σ_*β*_, σ_*β*0_, *τ*_*s*_, *ρ*, *ϕ*_0_, *ϕ*_*β*_). The mean effect of the parameters (*μ*_*β*_) had normal priors with a mean of 0 and a standard deviation of 10. I used Gamma priors with a shape of 1.2 and a rate of 0.2 for the standard deviation associated with each parameter (σ_*β*_), the standard deviation associated with the intercept (σ_*β*0_), and the heterogeneity parameters (*τ*_*s*_). For the correlation matrix among the parameters(*ρ*), I used a prior based on the C-vines approach, as defined in Lewandowski et al. [49], with a uniform prior on all correlation values. The spatial range for the intercept and the parameters (*ϕ*_0_, *ϕ*_*β*_) followed uniform priors, which were chosen to support a spatial range that ranged from a correlation of 0.01 at the smallest inter-strata distance to a correlation of 0.90 at the largest inter-strata distance. Models were fitted with the Hamiltonian Monte Carlo No-U-Turn sampler [50] using the Stan modelling language. I ran five chains with a burn-in period of 400 iterations, followed by 2000 iterations that were kept for analysis. Convergence was assessed using the split scale reduction factor [51] and visual inspection of the plotted chains. Simulations and posterior analyses were performed in R 3.0.2 [52] via the package rstan [53]. Figures were made with the package ggplot2 [54] in R 3.0.2.

### Derived parameters

A major advantage of MCMC analysis is that the derived parameters and their Credible Intervals (CI) can be estimated directly from the parameters of the model. For spatial correlation models, the effective range (K = 0.05) is often a more meaningful value than the range itself [55] and can be derived from $K_{0.05}=\sqrt{-\text{ln}\left(0.05\right)/\phi^{2}}$. The proportion of the variance in pond counts, explained by a given explanatory variable, can also be derived from estimated model parameters as in

$$p_{s,p}=\frac{{Β}_{s,p}{}^{2}\sigma_{s,p}^{2}}{\sum_{k=1}^{p}{Β}_{s,k}{}^{2}\sigma_{s,k}^{2}+\;\tau_{s,k}^{2}}$$ (8)

where $\sigma_{s,p}^{2}$ is the variance associated with explanatory variable *p* during the study period. The derived parameters do not contribute to the likelihood, but their posterior distributions reflect all of the uncertainties associated with the estimated parameters from which they were derived.

## Results

### Mean effects

The intercept, which represents the mean pond abundance in each strata, showed the greatest spatial variation. However, the intercept mean was at much larger scale than the mean of the predictors making comparison across the variables difficult. Precipitation in summer, winter, and fall, the number of ponds during the previous year, and spring precipitation presented the greatest spatial variation among the predictors (Table 1). The effect of temperature and time trends had much lower spatial variance. There were no significant correlations among the predictors (S2 Fig). However, the effects of the temporal trend had a tendency to be negatively correlated with the effects of precipitation in the summer, fall, and winter. These seasonal precipitation effects also tended to be positively correlated with each other. The effective range of the intercept tended to be larger (688; 95% Credible Intervals = 772–609) than the one governing the explanatory variables (644; 95% Credible Intervals = 983–483) although the 95% Credible Intervals overlapped (Table 1; S3 Fig). In both cases, however, the effective range is far greater than the mean nearest neighbour distances among strata ($\bar{\text{x}}$ = 250 km; sd = 106.83), indicating that the drivers in pond abundance were correlated at a broad scale.

**Table 1:** Summary for the mean effect (*μ*) of explanatory variables, the associated standard deviation(σ).

Parameter	Mean Posterior Distribution		Standard deviation Posterior Distribution	
	Median (50%)	Credible Interval (2.5–97.5%)	Median (50%)	Credible Interval (2.5–97.5%)
Intercept	11.931	(10.070–13.713)	7.390	(5.264–11.005)
Lag	0.259	(0.096–0.418)	0.144	(0.073–0.297)
Time	0.004	(-0.004–0.012)	0.007	(0.004–0.014)
Precipitation summer	0.343	(0.082–0.585)	0.206	(0.094–0.423)
Precipitation fall	0.251	(0.037–0.420)	0.182	(0.096–0.365)
Precipitation winter	0.372	(0.179–0.614)	0.189	(0.092–0.382)
Precipitation spring	0.315	(0.315–0.459)	0.112	(0.03–0.288)
Temperature summer	-0.020	(-0.051–0.022)	0.024	(0.005–0.062)
Temperature fall	-0.017	(-0.053–0.037)	0.036	(0.012–0.076)
Temperature winter	-0.010	(-0.026–0.010)	0.011	(0.003–0.031)
Temperature spring	-0.039	(-0.068–0.004)	0.033	(0.011–0.078)

### Spatial parameters

**Fig 2:**
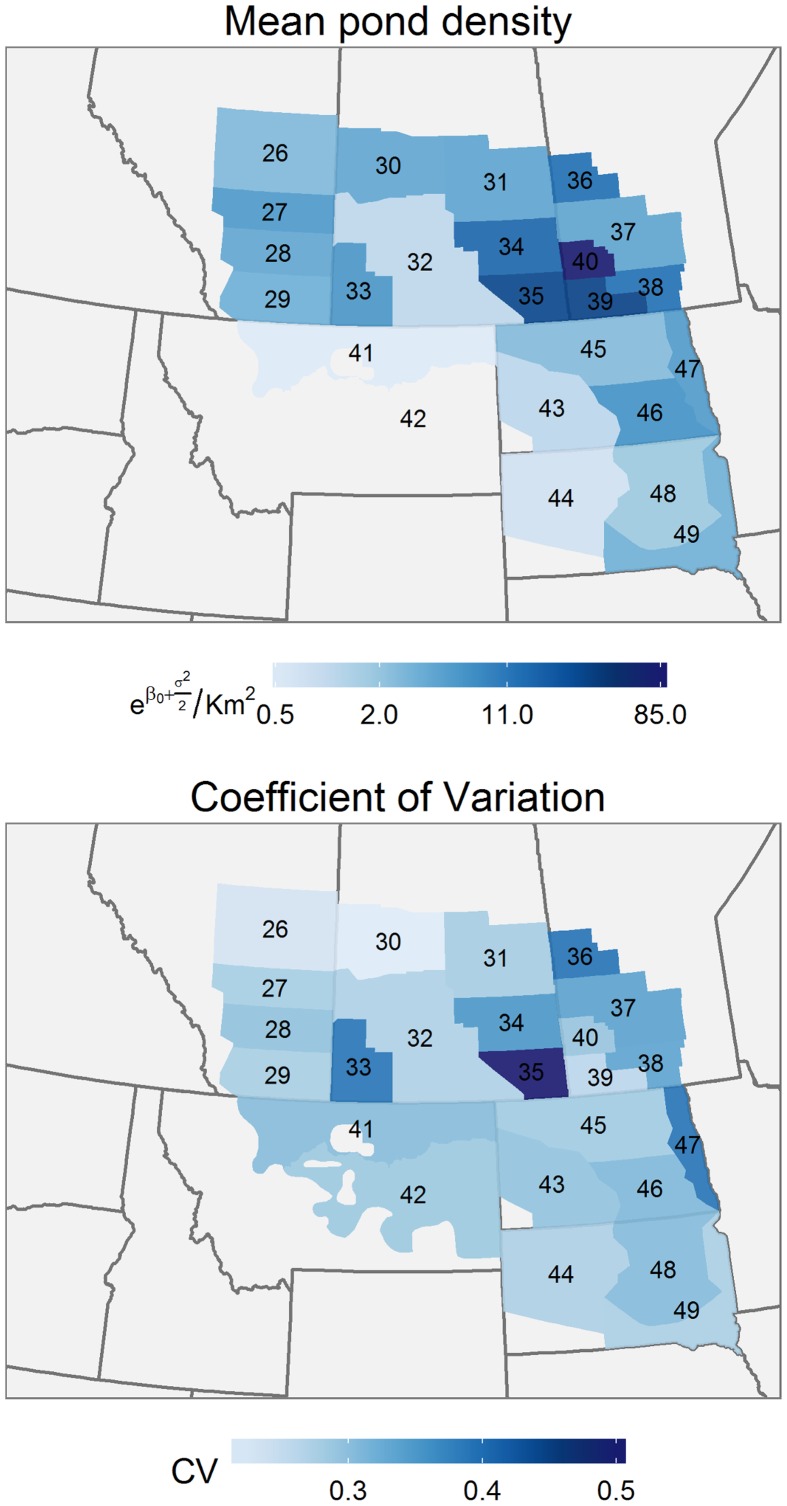
Spatial variation in mean pond density. Geographical variation in the stratum level, mean pond density ($e^{\beta_{0}+\frac{\sigma^{2}}{2}}/km^{2}$), and the predicted coefficient of variation ($\sqrt{e^{\sigma^{2}}-1}$) for pond abundance in the survey area.

**Fig 3:**
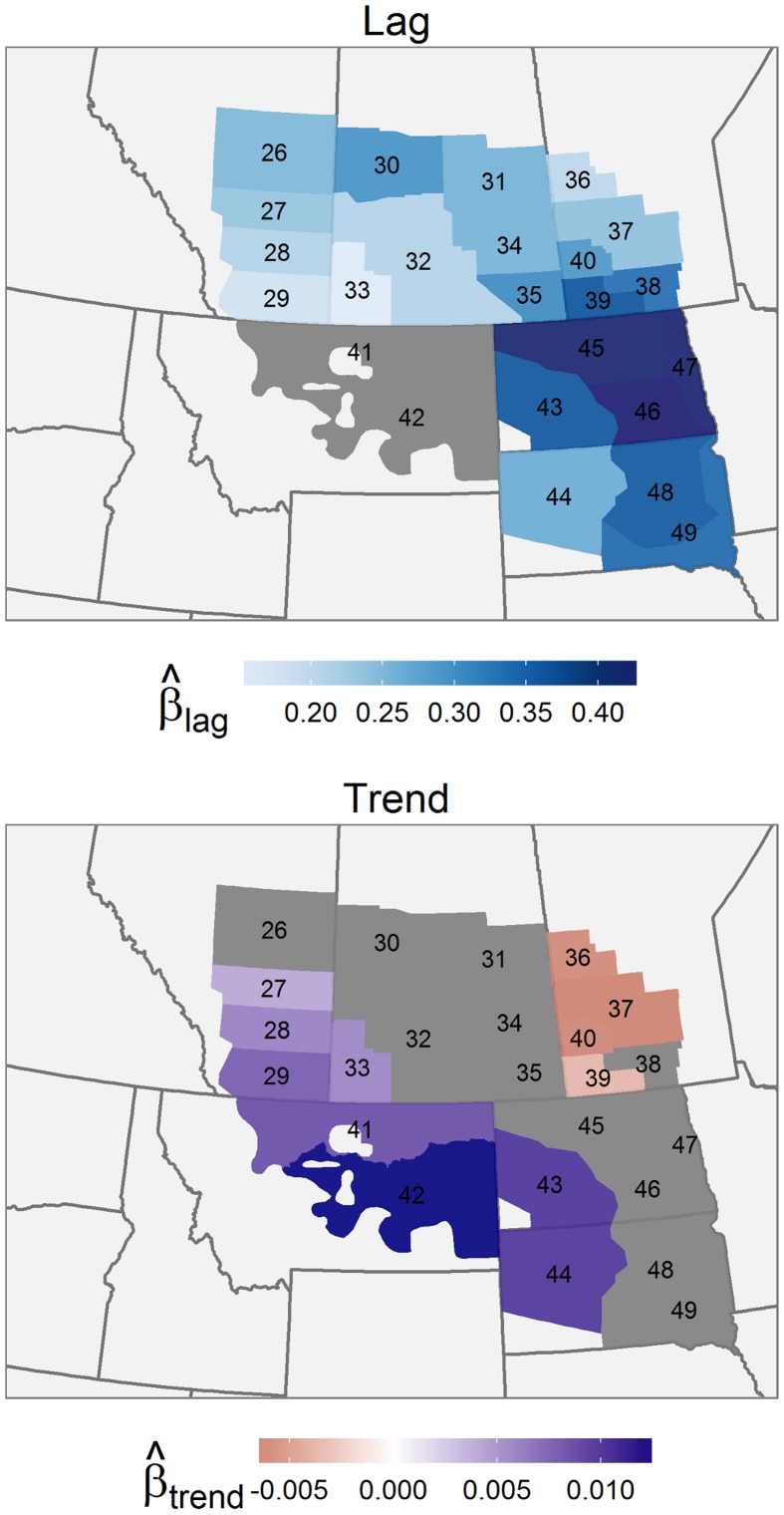
Effects of pond abundance in the previous year and temporal trends in pond abundance (1961–2012). Geographical variation in the estimated effects of pond abundance in the previous year (Lag) and the linear effect of time (Trend). In strata where the 95% Credible Intervals for a coefficient included 0, the effect is considered insignificant; these strata are coloured grey.

Mean annual pond density ($e^{\beta_{0}+\frac{\sigma^{2}}{2}}{\text{ km}}^{\text{-2}}$) was lowest in Montana and in southeastern Saskatchewan and highest in eastern Manitoba (Fig 2). The coefficient of variation in pond abundance ($\sqrt{e^{\sigma^{2}}-1}$) tended to increase towards the northeast quadrant of the survey area, indicating that pond abundance was proportionally more variable in this region. The lagged effect of pond density was greatest in the eastern half of the Dakotas (Fig 3) and lowest in Montana and southern Alberta. There was a strong north-east gradient in estimated pond abundance (Fig 3). Pond density in the southwest of the survey area increased significantly, but decreased significantly in Manitoba.

**Fig 4:**
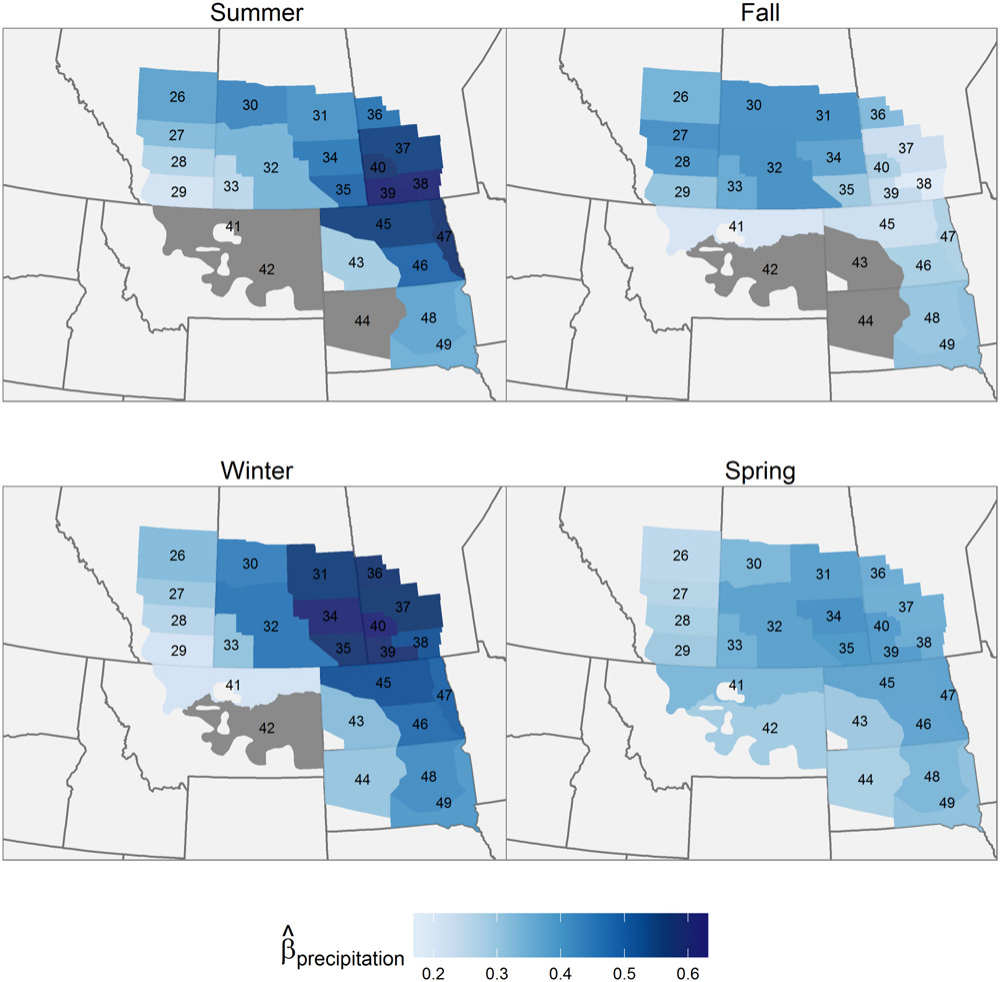
Effects of seasonal precipitation on pond abundance (1961–2012). Geographical variation in the predicted effect of seasonal precipitation during the previous year on pond abundance. In strata where the 95% Credible Intervals for a coefficient included 0, the effect is considered insignificant; these strata are coloured grey.

**Fig 5:**
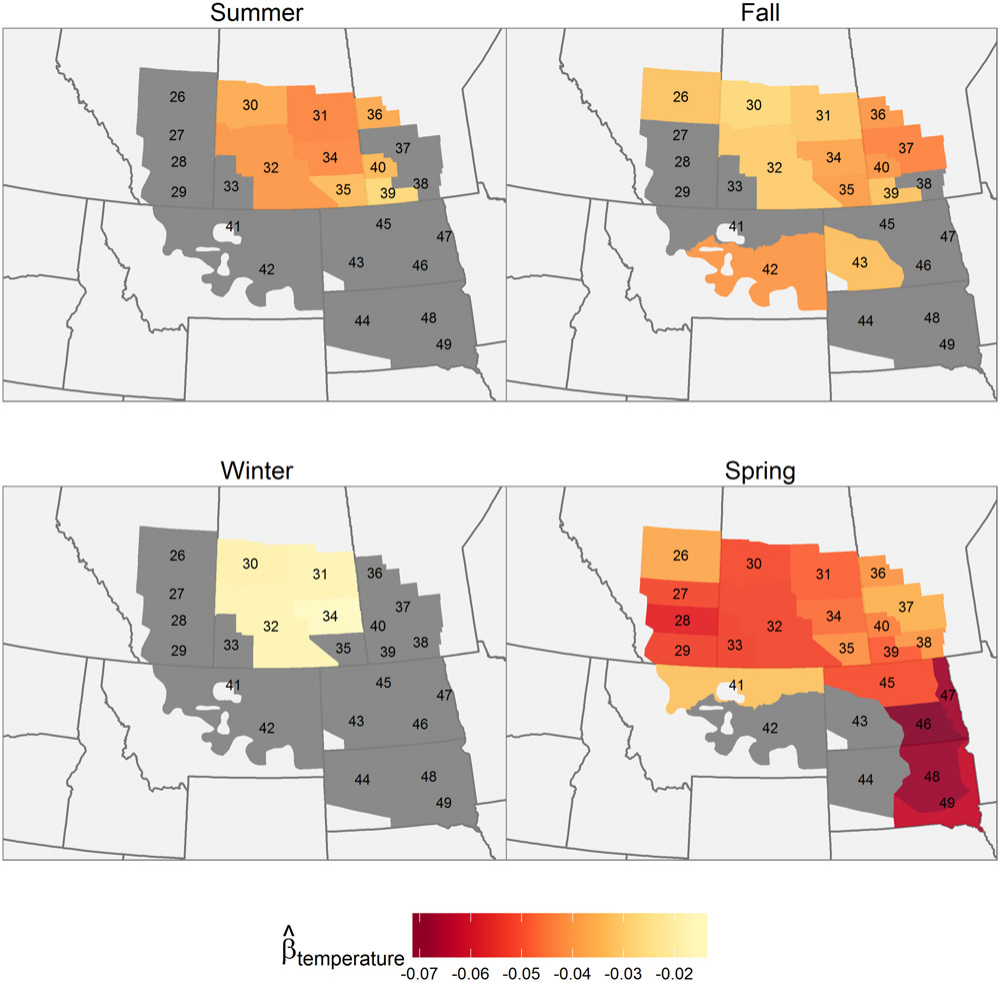
Effects of seasonal temperature on pond abundance (1961–2012). Geographical variation in the predicted effect of seasonal maximum temperature during the previous year on pond abundance. In strata where the 95% Credible Intervals for a coefficient included 0, the effect is considered insignificant; these strata are coloured grey.

All precipitation effects were positive. Saskatchewan experienced the greatest precipitation effects seasonally, with strong effects occurring there in summer, winter, and spring (Fig 4). Manitoba had strong precipitation effects in summer, fall, and winter. Fall precipitation also had a strong effect in Alberta. When looking across seasons, summer and winter precipitation exerted the strongest effects, particularly in the east, while fall and spring precipitation had a more limited effect. Summer temperatures negatively affected annual pond density in Saskatchewan (Fig 5), while fall temperature negatively affected pond density in the northern part of the survey area. Effects of winter temperature were minimal. Spring temperature was the most important among the four seasonal temperature-related variables, having a negative effect in all strata except in the western Dakotas and southern Montana.

**Fig 6:**
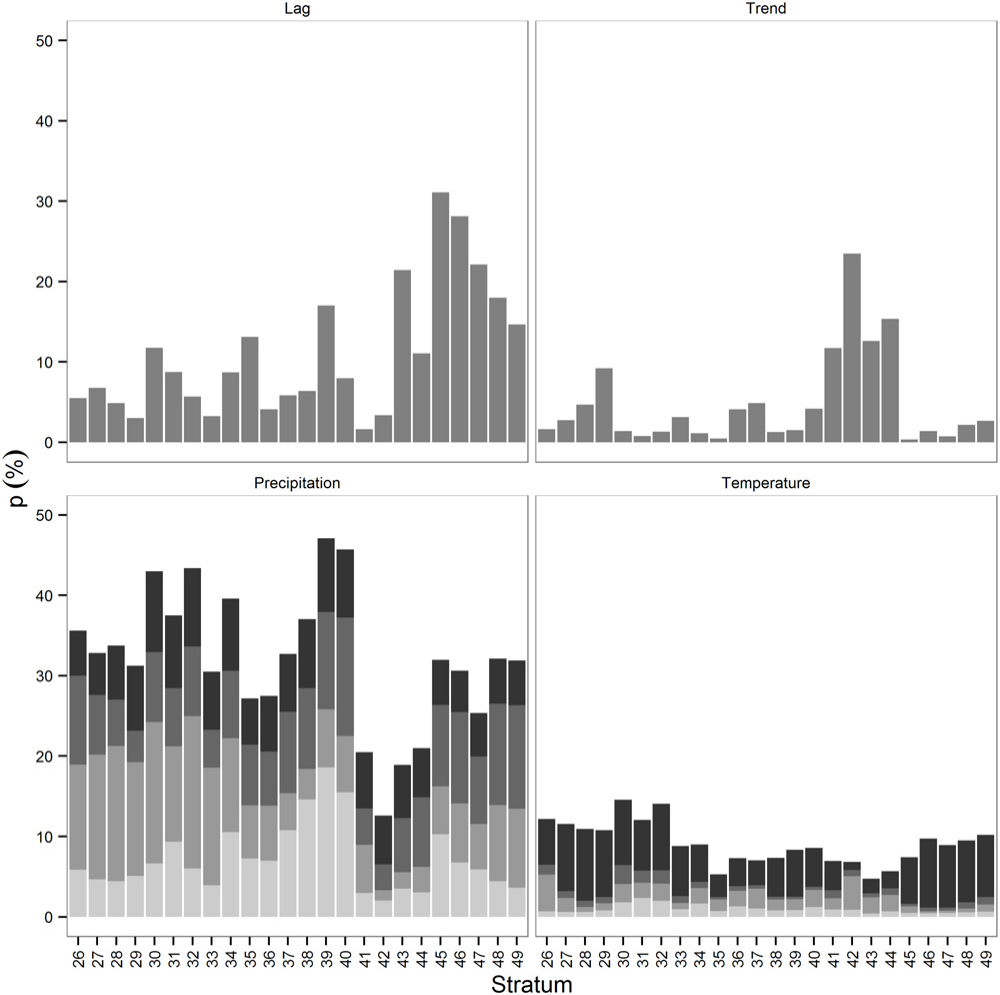
Proportion of within-pond variance explained by explanatory variables (1961–2012). Proportions of the within-strata pond variance during the survey period explained by the different explanatory variables. Seasonal precipitation and temperature are stacked from summer (light grey, bottom) to spring (black, top).

Lagged pond density explained 2–31% percent of all within-strata variance (Fig 6). The effect was, however, generally below 15%, with the exception of the strata in the eastern Dakotas. The proportion of variance explained by the temporal trends ranged between 0.4–23%, but explained less than 5% of the variance in the majority of strata. The combined effects of seasonal precipitation explained a greater proportion of the variance (12–46%) than the other variables. Summer precipitation explained the most variance for the strata in Manitoba, fall precipitation explained the most variance in Alberta and Saskatchewan, winter precipitation explained the most variance in the Dakotas, and spring precipitation was the most important driver in Montana. Overall, fall and winter precipitation explained the most variance across strata, followed by summer and spring precipitation, respectively. The combined effect of seasonal temperature explained only 3–15% of the variance, mostly attributed to spring temperature. Overall, pond dynamics in the survey area can be divided into four major classes. Ponds associated with the Great Plains in Montana and the western Dakotas were more dependent on spring and fall temperature. Ponds associated with the Prairies Pothole in the eastern Dakotas were dependent on the abundance of ponds during the previous year and on winter precipitation. The ponds in the Parklands in Manitoba and eastern Saskatchewan were more closely tied to summer and winter precipitation, whereas ponds in Alberta and western Saskatchewan were more affected by fall and spring temperature.

## Discussion

As expected, there was considerable spatial heterogeneity in the drivers of pond abundance across the survey area, most notably in seasonal precipitation and lagged pond abundance. Pond abundance in the northwest of the survey area was most dependent on precipitation and temperature during the previous year, while the ponds in the southeast were more dependent on pond abundance during the previous year. Also as expected, pond abundance was significantly correlated with spring temperatures, but less so for the other seasons. However, estimated temporal trends were not according to expectations. Pond abundance in the northeast has been steadily declining, which I predicted based on the results of Watmough and Schmoll [16]. However, contrary to my expectations, pond abundance has been increasing in the southwest part of the survey area.

### Temporal lag and precipitations

Ponds in the northwest are more dependent on climatic variables, while the wetlands in the southeast are more dependent on pond previous year conditions. This spatial pattern in the dependence on lagged pond abundance matches the observed variation in wetland stability reported previously in the scientific literature [18]. Wetlands in the east are generally believed to be deeper and thus more stable [56]. Winter and fall precipitation were the main drivers for most strata in the east, in accordance with previous studies [12,14]. However, spring and summer precipitation during the previous year explained a respectable proportion of the variance in pond abundance (≥8.10%), particularly in the west. Contributions of spring and summer precipitation has generally been overlooked by previous studies of this dataset (but see [56]). Seasonal precipitation was not only important in terms of temporal abundance, but was also among the key drivers of the spatial variation in the model. This is at odds with Larson’s report [12] of low spatial variation in precipitation effects. Her analysis was restricted to the PPR and did not include all four seasons, which may explain the discrepancy. My results, however, underline that there is considerable heterogeneity in the precipitation drivers in different regions of the survey area. Thus, using a single parameter for each variable contributing to pond quantity dynamics, as has been done in the past, might yield misleading results. From a statistical viewpoint, using such a model could lead to a situation where the model predictions represent an average that does not appear anywhere in the study area [26]. Moreover, from a purely utilitarian perspective, addressing non-stationarity will improve model fit and model prediction [34]. This final point is particularly important if we want to accurately forecast the future behavior of the system.

### Temperature

Overall, the effect of seasonal temperature was generally weak compared to other variables. This suggests seasonal temperatures are a weak regulator of pond abundance, at least within the range of climatic conditions observed in our study. Spring was the most important season, and its importance was greatest in the south. The importance of spring temperatures may be explained by water behavior on frozen surfaces. Precipitation falling on frozen ground should persist rather than percolate into the soil, thereby increasing pond size and abundance [12]. A warm spring would thus lead to a decrease in pond size and abundance. Fall temperatures had a limited impact in the north, and in general, the effect of fall temperatures should be similar to the effect of spring temperatures. Spring and fall temperatures were reported by Larson [12] as important factors in pond abundance, but did not report the effect of summer temperature as she did not test for it. My results indicate that winter temperature effects were negligible, and that summer temperatures have only a weak effect in the center of the PPR. Indeed, the effects of summer temperatures are straightforward: higher temperature leads to higher evapotranspiration, which in turn diminishes pond size and abundance.

### Temporal trend in pond abundance

There is a clear northeast-southwest gradient in the temporal trend in pond abundance. Increasing trends mostly occurred in southeastern strata, while decreasing trends were confined to the northeast. This is consistent with Niemuth et al [14]. However, their work did not account for annual variation in weather; a good deal of the increase they observed could have reflected wetter conditions during the survey period [5]. The trends I report account for climate, and so may represent increases due to non-climatic factors not included in the model. As such, my results may be a better representation of the impact of land conversion for agricultural or other anthropogenic purpose, change in agricultural practices, and the effects of the various conservation efforts that have been deployed in the area to protect and restore wetlands during the study time frame.

For example, increased pond abundance in the USA strata could be related to the various conservation efforts of the U.S. Department of Agriculture. The Reserve Program and Wetlands Reserve Program together have contributed to the restoration of 0.2 million ha of wetland and grassland habitats in the PPR [1]. While these results should be seen as encouraging, the future of wetland conservation efforts in the USA is uncertain. In particular, payments made to farmers participating in conservation programs have failed to keep pace with rising values of agricultural commodities [57].

In Canada, the decreasing abundance I report for Manitoba ponds corroborate the results of Watmough and Schmoll [16]. They also detected a decline in ponds in Alberta, whereas I detected an increase in ponds. The discrepancy could be partially due to differing timescales. Watmough and Schmoll [16] studied only a subset (1987–2000) of the time period I used. My results appear to indicate that wetland conservation and enhancement efforts in Alberta performed better than those in Manitoba. This is consistent with a disproportionate amount of the Prairie Habitat Joint Venture's effort taking place in Alberta in response the dramatic declines in duck abundance in this province [56]. Agricultural expansion has also been historically more extensive in the east than in the west in the Canadian Prairies, and the agricultural practices in the east have also been more prone to degrade wetlands habitat [56]. The decline observed in Canada are also probably explained in part by the fact that wetland conservation and monitoring are, for the most part, under provincial jurisdiction; as a result, there is no large-scale Canadian wetlands conservation program, such as the United States' WRP. Given that the Prairie Habitat Joint Venture coordinates many of the major wetland conservation and enhancement efforts in the survey area, some effort could be made to consolidate conservation programs across provinces and step up conservation efforts in the eastern part of the Prairies.

### Implications for climate change

Until now, most efforts to forecast climate change impacts using the WBHS data have used analytical approaches that do not capture the spatial heterogeneity inherent in the region. However, spatial heterogeneity in climate can represent an important spatial buffer, which affects regional response to climate change [58]. Indeed, climate change did not occur uniformly across the PPR during the twentieth century [5]. The Great Plains have warmed considerably, but the increase was lower in the Parkland, and temperature actually declined in the southeastern PPR [5]. In light of my results, if the spatial patterns seen in past temperature increases persist, impacts of climate warming on pond abundance in the spring could be less dramatic than previously thought. The areas that are predicted to grow warmer are those where pond abundance appear to be the least dependent on temperature, while the areas that are cooling are the most sensitive to temperature. The seasonal timing of these changes will be crucial, however. For example, Johnson et al. [19] suggested that warmer winters could increase sublimation losses, reducing snowpack, and therefore water levels and pond abundance in the spring. This is a pertinent consideration, since the IPCC [59] projects winters will warm more than summers in many regions. My analysis suggests, however, that winter temperature had a relatively minor effect on wetland abundance in the spring in the PPR. It follows that unless winter temperatures exceed the levels observed during my study period, an increase in temperature in winter would be less deleterious for water levels and wetland abundance than a corresponding increase in spring temperatures.

A lot of the uncertainty surrounding predicted impacts of climate change on wetland dynamics stems from the fact that predictions for precipitation are still imprecise [59]. Although the PPR and the GP became wetter during the twentieth century [5,60], the region is known to go through extended wet-dry periods [5]. There is some evidence of changing seasonality, with precipitation now occurring more in early summer and fall [60]. Should this trend continue, an increase in fall precipitation would actually occur during a key period for ponds in the northwest, precipitation which could potentially offset the negative effect of an increase in temperature. However, recent increases in precipitation have been more pronounced in the east, where wetlands are less dependent on precipitation, than in the west [5,18,19]. Any speculation about future spring abundance of wetlands will be constrained by the accuracy of climatic forecasts for the region. Current climate projections for precipitation in the study area range between a 5% decline to a 10% increase [59]. Given that precipitation is the main driver of wetland abundance in the spring, making long-term predictions will be impossible unless we can derive more accurate long-term forecasts of precipitation.

### Limitations of the dataset

The WBPHS pond data allow us to look at the dynamics of wetlands as measured in May, and does not distinguish wetland type. Temporary, seasonal, and semi-permanent wetlands all have different dynamics and play different roles in the ecology of waterfowl and migrating birds [19,61]. Because these types are not distinguished in the survey, my results represent an average response. Secondly, the waterfowl brooding and breeding period of many waterbirds extend into summer. There is mounting evidence that summer wetland conditions are important limiting factors for waterfowl populations, and the pond surveys may not provide an adequate measure of habitat quality throughout the year. It is increasingly understood that the prevalence and persistence of summer wetlands are important factors for waterfowl populations [62]; spring pond surveys, therefore, may not provide an adequate measure of habitat quality over all seasons.

## Conclusion

In this paper, I argue for the importance of considering spatial heterogeneity when modeling wetland dynamics. I demonstrated that various regions and sub-regions may exhibit a range of responses to climactic influences by assessing a 50-year dataset using a SVC-based model. Identifying the underlying processes responsible for the spatial heterogeneity observed allow us to develop stronger inference about the ecosystem of interest [23]. Efforts to assess the impact of climate and land use changes through large-scale modelling require an intimate knowledge of an ecosystem and the heterogeneity within it in order to take into account regional variability. Developing this knowledge will require allocating significant resources to well-designed spatially extensive monitoring programs, like the WBPHS. Such programs must be sufficiently adaptable to respond to new, or newly identified, information needs. Perhaps most importantly, ecologists aiming to forecast climate change impacts will need to fully embrace both the challenges associated with spatial heterogeneity and the tools that will allow them to model it.

## Supporting Information

S1 Fig Geographical variation in annual precipitation and temperature (1961–2012). Mean and variance of the total annual rainfall and average maximum temperature as a function of seasons for each strata between 1961 and 2010.
(TIFF)

S2 Fig Correlation among predictors. Each rectangle presents the posterior distribution of the correlation parameter between the predictors. Plotting areas range between -1, and 1, the dashed grey lines indicates 0, and the black lines represent the median estimate and the upper and lower 95% Credible Intervals. None of the estimated correlations are significantly different from 0.
(TIFF)

S3 Fig Spatial correlation function. Predicted spatial correlation function (black line) with the 95% Credible Intervals for the intercept (K_0_) and the explanatory variables (K_B_). The dotted line represent the effective range (K = 0.05)
(TIFF)
