# Supplementary figures and images for: Quantifying Geographic Variation in the Climatic Drivers of Midcontinent Wetlands with a Spatially Varying Coefficient Model
Source: PLoS One. 2015 Apr 27;10(4):e0126961. doi: 10.1371/journal.pone.0126961 (PMC4411070; doi:10.1371/journal.pone.0126961)

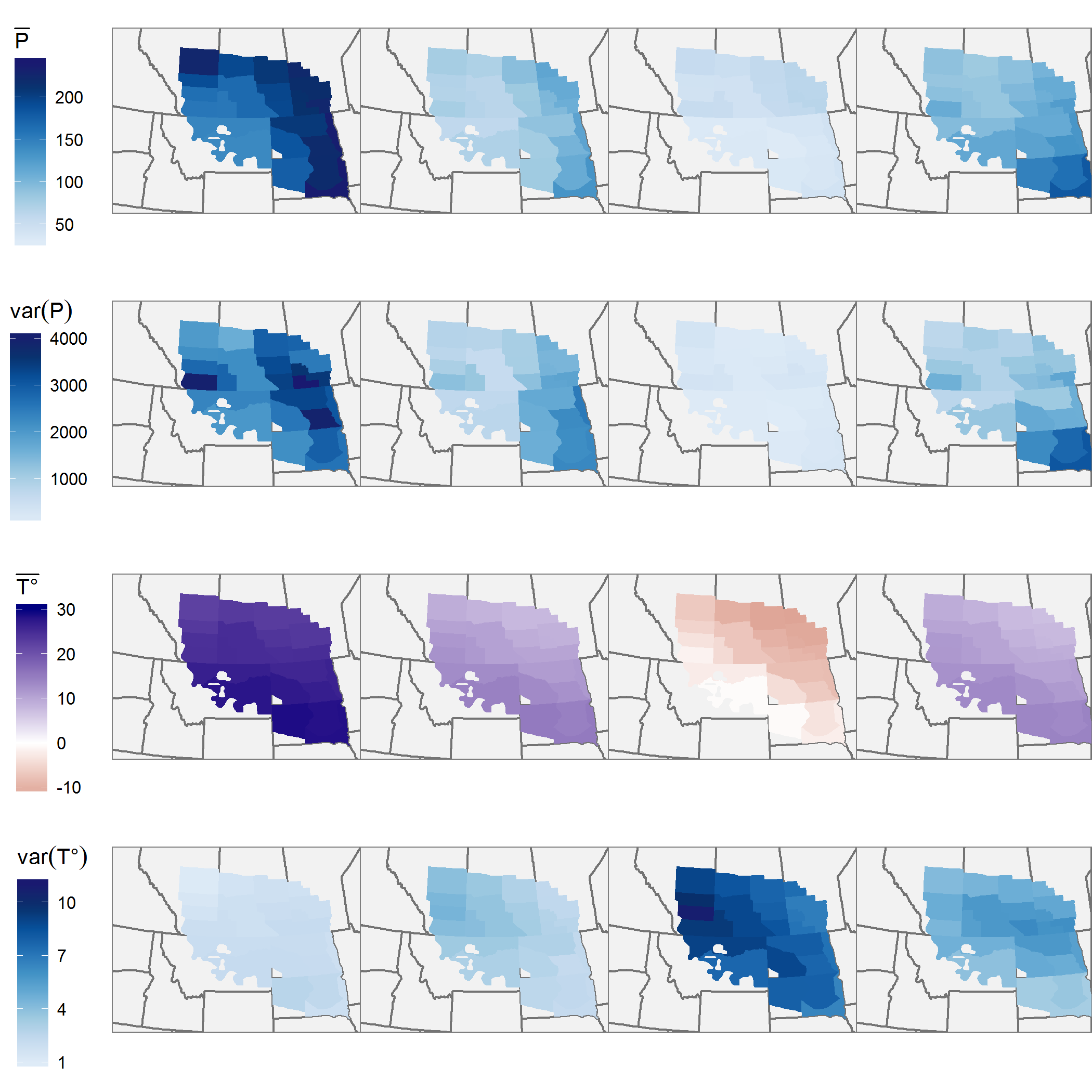

Supplement: S1 Fig — Mean and variance of the total annual rainfall and average maximum temperature as a function of seasons for each strata between 1961 and 2010. (TIFF) [file pone.0126961.s001.tiff]

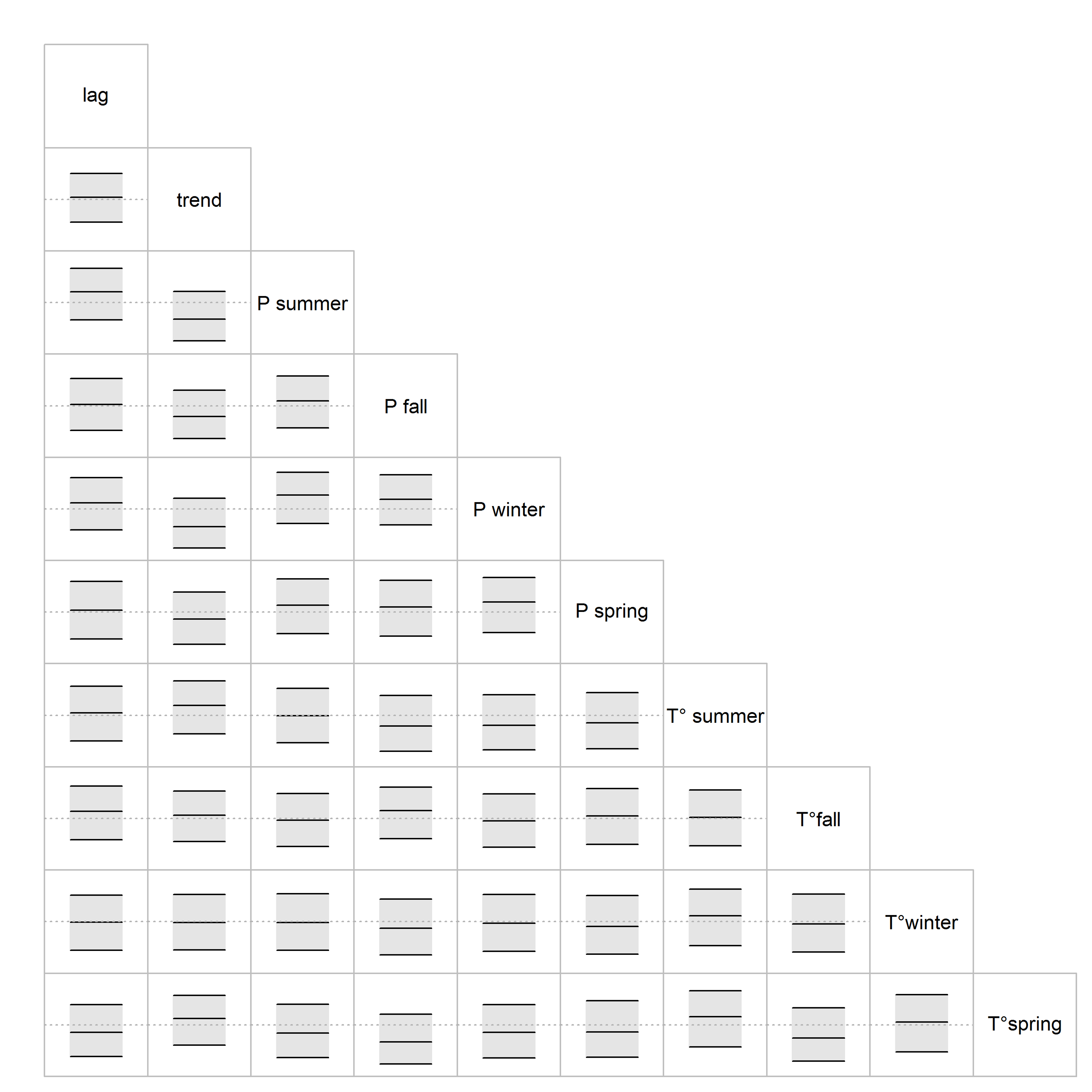

Supplement: S2 Fig — Each rectangle presents the posterior distribution of the correlation parameter between the predictors. Plotting areas range between -1, and 1, the dashed grey lines indicates 0, and the black lines represent the median estimate and the upper and lower 95% Credible Intervals. None of the estimated correlations are significantly different from 0. (TIFF) [file pone.0126961.s002.tiff]

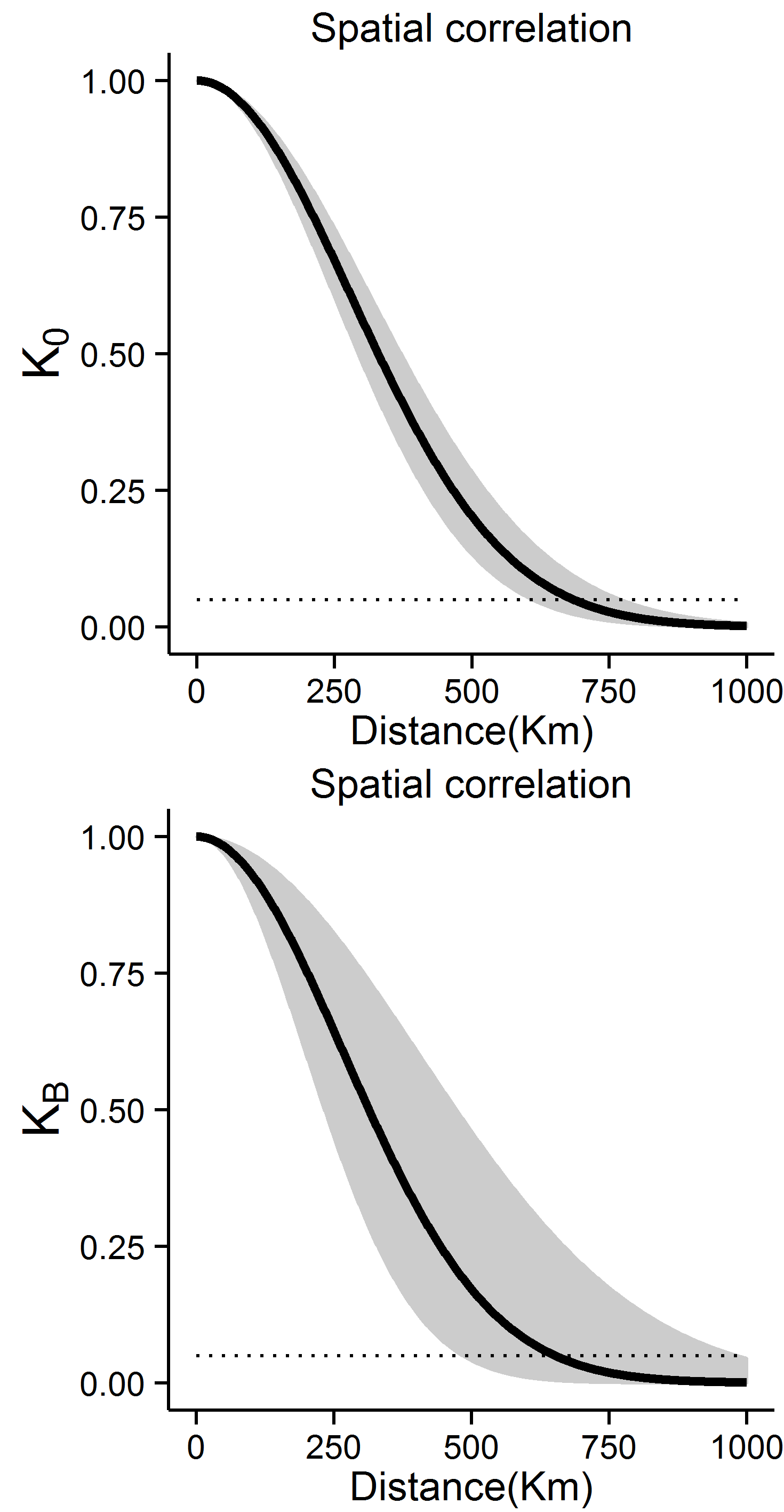

Supplement: S3 Fig — Predicted spatial correlation function (black line) with the 95% Credible Intervals for the intercept (K0) and the explanatory variables (KB). The dotted line represent the effective range (K = 0.05) (TIFF) [file pone.0126961.s003.tiff]
